# Regenerative therapies for refractory thin endometrium in *in vitro* fertilization

**DOI:** 10.3389/fcell.2025.1668960

**Published:** 2025-10-24

**Authors:** Hsuan-Ju Chen, Ming-Jer Chen, Po-Han Chang, Yu-Ting Lu, Ya-Wen Hsueh, Chia-Wei Chang, Hsi-Chen Hsu, Tung-Chuan Yang, Wu-Chou Lin, Maw-Sheng Lee, Hsun-Ming Chang

**Affiliations:** ^1^ Department of Obstetrics and Gynecology, China Medical University Hospital, Taichung, Taiwan; ^2^ Division of Infertility, Lee Women’s Hospital, Taichung, Taiwan

**Keywords:** thin endometrium, IVF, regenerative therapies, PRP, G-CSF, growth hormone, stem cells, exosome

## Abstract

Refractory thin endometrium remains a significant challenge in assisted reproductive technology, frequently leading to poor endometrial receptivity and suboptimal outcomes in in vitro fertilization (IVF) cycles. In recent years, regenerative therapies such as platelet-rich plasma (PRP), granulocyte colony-stimulating factor (G-CSF), growth hormone (GH), and stem cell-based interventions, have gained increasing attention as promising strategies to enhance endometrial function and receptivity. Notably, growing interest has also focused on the therapeutic potential of stem cell-derived exosomes in facilitating endometrial repair, although clinical evidence remains limited. Recent studies suggest that regenerative interventions are administered either locally, *via* intrauterine infusion or sub-endometrial injection, or systemically at various stages of the IVF protocol. Among these approaches, PRP and G-CSF have shown consistent benefits in increasing endometrial thickness and improving clinical pregnancy rates, particularly in frozen embryo transfer cycles. GH has been associated with enhanced endometrial proliferation, while stem cell-based therapies, particularly those utilizing mesenchymal or bone marrow-derived stem cells, demonstrate potential to restore severely damaged endometrial tissue. In preclinical models, stem cell-derived exosomes have been shown to promote endometrial regeneration and angiogenesis, underscoring their potential for future clinical application. Despite these encouraging developments, most studies are constrained by small sample sizes, methodological heterogeneity, and variable treatment protocols, which hinder the ability to draw definitive conclusions. Taken together, regenerative therapies represent a promising new direction in managing refractory thin endometrium among IVF patients. Preliminary clinical outcomes, particularly those associated with PRP, G-CSF, GH, and stem cell-based approaches, are encouraging. However, robust, large-scale, and well-controlled clinical trials are crucial for validating efficacy, optimizing therapeutic protocols, and ensuring long-term safety. Among these innovations, stem cell-derived exosomes stand out as an especially exciting and emerging frontier in reproductive medicine, supported by compelling preclinical evidence that merits further investigation.

## 1 Introduction

Infertility affects approximately 17.5% of the adult population worldwide, posing significant physical, emotional, and financial challenges for affected individuals and couples (Cox et al., 2022). The advancement of assisted reproductive technologies (ART) has provided effective solutions for various infertility conditions, with *in vitro* fertilization (IVF) being one of the most widely utilized treatments. IVF involves a complex sequence of procedures, including controlled ovarian stimulation, oocyte retrieval, fertilization, and embryo transfer. Despite improvements in IVF protocols, the success of implantation remains dependent on multiple factors, including embryo quality, endometrial receptivity, and synchronization between the embryo and the uterine environment (Ojosnegros et al., 2021).

Endometrial thickness is considered a critical determinant of endometrial receptivity and implantation success. A sufficient endometrial lining provides an optimal environment for embryo implantation, whereas a thin endometrium has been associated with lower pregnancy rates and higher miscarriage risks (Revel, 2012). Numerous studies have demonstrated that inadequate endometrial thickness negatively impacts clinical pregnancy and live birth outcomes in both natural and ART cycles (El-Toukhy et al., 2008; Bozdag et al., 2009; Kasius et al., 2014). However, there is no universal consensus on the definition of thin endometrium, with most studies considering it to be an endometrial thickness of less than 6–8 mm during the secretory phase (Rodríguez-Eguren et al., 2024).

The management of thin endometrium remains a challenge in reproductive medicine. Various therapeutic approaches have been investigated to improve endometrial thickness and receptivity, including extended estrogen administration (Shen et al., 2013; Chen et al., 2006), low-dose aspirin (Hsieh et al., 2000), sildenafil citrate (Li et al., 2020), and pentoxifylline-tocopherol combination therapy (Krief et al., 2021; Acharya et al., 2009). While some patients respond favorably to these interventions, others fail to achieve optimal endometrial growth, leading to recurrent implantation failure and cycle cancellations. This highlights the need for novel therapeutic strategies that can effectively promote endometrial regeneration in refractory cases.

**FIGURE 1:**
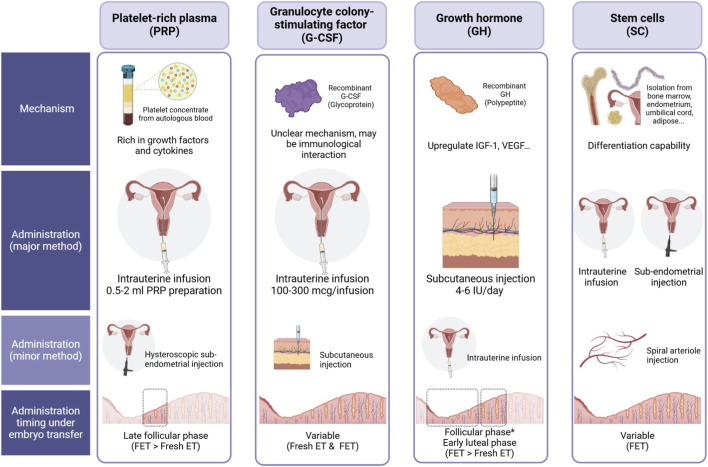
Comparison of four biological therapies—Platelet-Rich Plasma (PRP), Granulocyte Colony-Stimulating Factor (G-CSF), Growth Hormone (GH), and Stem Cells (SC)—for endometrial enhancement in assisted reproductive technology (ART). The figure outlines their proposed mechanisms of action, administration routes, and optimal timing relative to embryo transfer. • **PRP**: Derived from autologous blood, PRP is rich in growth factors and cytokines. It is primarily administered *via* intrauterine infusion (0.5–2 mL) and can also be delivered through hysteroscopic endometrial injection. The recommended timing is during the late follicular phase, with a preference for frozen embryo transfer (FET) over fresh embryo transfer (Fresh ET). • **G-CSF**: A recombinant glycoprotein with a potentially immunomodulatory mechanism. The primary administration method is intrauterine infusion (100–300 μg), while subcutaneous injection is an alternative approach. Its timing is variable, allowing use in both Fresh ET and FET cycles. • **GH**: A recombinant polypeptide that upregulates IGF-1 and VEGF, potentially improving endometrial receptivity. It is predominantly administered *via* subcutaneous injection (4–6 IU/day) but can also be delivered intrauterinely. Administration typically occurs during the follicular and early luteal phases, with a preference for FET over Fresh ET. • **SC**: Stem cells, isolated from various sources (such as bone marrow, endometrium, umbilical cord, and adipose tissue), exhibit differentiation potential. They are administered *via* intrauterine infusion or sub-endometrial injection, with an additional option for spiral arteriole injection. Timing is variable, primarily favoring FET cycles.

In recent years, regenerative medicine has emerged as a promising avenue for enhancing endometrial growth and function. Several biological therapies, including platelet-rich plasma (PRP), granulocyte colony-stimulating factor (G-CSF), growth hormone (GH), stem cell therapy, and stem cell-derived exosomes, have been investigated for their potential to restore endometrial receptivity in women with thin endometrium (Figure 1). These approaches aim to harness the body’s natural healing mechanisms to stimulate tissue proliferation, angiogenesis, and immune modulation, ultimately improving implantation potential and pregnancy outcomes.

This literature review provides a comprehensive analysis of the clinical evidence supporting the use of PRP, G-CSF, GH, and stem cell therapy for the treatment of thin endometrium in women undergoing infertility treatment (Figure 1). Additionally, we explore experimental findings on the regenerative effects of exosomes derived from stem cells, offering insights into their potential role in endometrial repair and future therapeutic applications. By summarizing the latest advancements in regenerative therapy, this review aims to contribute to the development of innovative treatment strategies for women with refractory thin endometrium undergoing IVF.

To enhance transparency, we conducted a structured literature search to identify relevant studies on regenerative therapies for thin endometrium. Electronic databases, including PubMed, Embase, and Web of Science, were searched up to July 2025. The search strategy combined Medical Subject Headings (MeSH) and free-text terms such as “thin endometrium,” “endometrial receptivity,” “*in vitro* fertilization,” “platelet-rich plasma,” “granulocyte colony-stimulating factor,” “growth hormone,” “stem cell therapy,” and “exosomes.” Boolean operators (AND/OR) were applied to capture both broad and specific results. Inclusion criteria were: (1) clinical studies (randomized controlled trials, cohort studies, case series, and case reports) or preclinical studies (animal or *in vitro* experiments) evaluating PRP, G-CSF, GH, stem cells, or stem cell-derived exosomes in relation to endometrial thickness, receptivity, or IVF outcomes; (2) articles published in English; and (3) studies providing measurable outcomes relevant to implantation, pregnancy rates, or endometrial repair. Exclusion criteria included: (1) studies not focused on reproductive outcomes (e.g., oncology, unrelated regenerative contexts), (2) reviews, editorials, or conference abstracts without primary data, and (3) duplicate publications.

Given the narrative nature of this review, a formal risk-of-bias assessment (e.g., Cochrane RoB or Newcastle–Ottawa Scale) was not applied. However, study design, sample size, and methodological strengths/limitations were explicitly noted in the Results and Discussion sections to allow readers to critically assess the quality of evidence. This approach ensured a broad yet focused synthesis of the available literature, balancing comprehensive coverage with clinical relevance.

## 2 Platelet-rich plasma (PRP) therapy

The use of PRP in infertile women with refractory thin endometrium has shown promising results in promoting endometrial expansion. PRP facilitates tissue proliferation, repair, angiogenesis, and immunomodulation by delivering key growth factors, including vascular endothelial growth factor (VEGF), tumor growth factor-β (TGF-β), insulin-like growth factor-1 (IGF-1), platelet-derived growth factor (PDGF), and epidermal growth factor (EGF) (Mouanness et al., 2021). Despite its potential, no standardized protocol for PRP preparation has been established, although various methods have been reported. Most studies have utilized autologous PRP, derived from the patient’s own peripheral venous blood, with red blood cells removed through centrifugation (Table 1). Recently, a case series demonstrated the efficacy of lyophilized PRP administered *via* intrauterine infusion, reporting increased endometrial thickness (EMT) without adverse effects (Gangaraju et al., 2023).

**TABLE 1:** PRP treatment for refractory thin endometrium.

Study	Objective	Samples	Blood/PRP (mL)	PRP administration	Results (PRP vs control)
Case series and cohort study					
Chang et al. (2015) Case series	History of cycle cancelation due to thin EMT ( ≤ 7 mm)	5	15/0.5–1	Infusion on HRT D10; may repeat after 72 h if unsatisfied EMT (n = 4)	All reached adequate EMT (>7 mm) Clinical pregnancy (5/5)
Zadehmodarres et al. (2017) Case series	History of FET cancellation due to thin EMT (<7 mm)	10	17.5/0.5	Two infusions on HRT D11-12 and D13-14	• All reached EMT > 7 mm after two infusions • Positive pregnancy tests (5/10) • Clinical pregnancy (4/10)
Molina et al. (2018) Prospective cohort	At least one failed cycle; history of thin EM	19	NM/1	Two infusions on HRT D10 and D12	• All reached EMT >7 mm after the 1st infusion; >9 mm after the 2nd • Positive pregnancy test: 14/19 (73.7%) • Ongoing pregnancy and live birth: 5/19 (26.3%)
Kim et al. (2019) Prospective cohort	History of ≧ 2 failed cycles; refractory thin EM	24	30/0.7–1	Infusion from HRT D10 every 3 days until EMT reached 7 mm (max 3 times)	• Compare to the previous cycle Increase of EMT (mean 6 mm), without significance Significant increase in IR (12.7%), CPR (30%), and LBR (20%)
Agarwal et al. (2020) Retrospective, cross-section	History of FET cancellation due to thin EMT (<7 mm) despite increasing E2, PRP infusion, or G-CSF	32	8/4	Sub-endometrial injection under HRT	• EMT >7 mm: 24/32 (75%) • Clinical pregnancy: 10/24 (41.66%) • Live birth: 5/24 (20.83%)
Frantz et al. (2020) Case series	EMT <5 mm after 14–17 days of E2 usage	21 (24 cycles)	NM/0.5	Infusion every second day for 3 times	• Clinical pregnancy: 16/24 (66.7%) • Ongoing pregnancy or live birth: 13/24 (54%)
Dogra et al. (2022) Prospective cohort	History of cycle cancellation due to thin EM (<7 mm)	20 (26 cycles)	15/0.5–1	Infusion on HRT/stimulation D8; repeated every 48 h until EMT >7 mm (max 3 times) Fresh ET (14 cycles); FET (12 cycles)	• Significantly increased EMT after PRP (p < 0.001) Fresh ET: from 5.83 to 7.14 mm FET: from 5.52 to 7.14 mm • LBR: 33.3% (fresh) and 9.1% (FET)
Lin et al. (2023) Retrospective cohort	RIF and thin EM (<7 mm)	77	20/1	Two infusions at 48 h intervals on HRT D11-13	• Significantly increased EMT after PRP: from 6.4 to 8.3 mm • IR, CPR, LBR: 21%, 33%, 21%
Controlled trial					
Eftekhar et al. (2018) RCT	Poor endometrial response (EMT <7 mm) to HRT	83	8.5/1.5	PRP (n = 40): infusion on HRT Day 13; repeat if EMT < 7 mm after 48 h Control (n = 43): HRT only	• Significantly thicker EM in the PRP group: 8.67 vs 8.04 mm (p = 0.001) • Significantly higher CPR in the PRP group: 32.5% vs 14% (p = 0.044) • No difference in ongoing pregnancy rate
Nazari et al. (2019) Double-blind RCT	History of FET cancellation due to thin EM (<7 mm)	60	17.5/0.5	Infusion on HRT D11-12; repeated after 48 h as needed PRP (n = 30): IUI catheter Control (n = 30): sham catheter	• Significantly thicker EM in the PRP group: 7.21 vs 5.76 mm (p < 0.001) • Significantly higher CPR in PRP group: 33.3% vs 3.3% (p = 0.048)
Chang et al. (2019) Prospective cohort	History of cycle cancellation due to thin EM (<7 mm)	64	15/0.5–1	PRP (n = 34): infusion on HRT D10 and P day Control (n = 30): HRT only	• Significantly thicker EM in the PRP group: 7.65 vs 6.52 mm (p = 0.013) • Significantly higher IR and CPR in the PRP group IR: 27.94% vs 11.67% (p = 0.018); CPR: 44.12% vs 20% (p = 0.036)
Kusumi et al. (2020) Prospective single arm-controlled	RIF and thin EM (<7 mm)	36	20/1	First cycle: HRT only 2nd cycle: PRP infusion on HRT D10 & 12	• Significantly thicker EMT on D14 in the 2nd cycle Unblinded: from 5.98 to 7.25 mm Blinded: from 6.04 to 6.76 (p < 0.001)
Yu et al. (2024) Prospective case-controlled	History of failed euploid FET cycle and thin EM (<7 mm)	116	20/2	PRP treatment on HRT D11-13 Infusion (n = 55): twice at 48 h interval Sub-endometrial injection (n = 38) Control: (n = 23): HRT only	• Significantly thicker EM in PRP groups (p < 0.001) Infusion: 8.8 mm; sub-endometrial: 8.7 mm; control: 6.7 mm • Significantly higher IR and LBR in sub-endometrial group than control IR: 52% vs 18% (p < 0.001) LBR: 38% vs 4% (p = 0.0082)

Abbreviations: PRP, platelet rich plasma; EMT, endometrial thickness; HRT, hormone replacement therapy; FET, frozen embryo transfer; EM, endometrium; NM, not mentioned; IR, implantation rate; CPR, clinical pregnancy rate; LBR, live birth rate; E2, Estradiol; G-CSF, granulocyte colony-stimulating factor; RCT, randomized controlled trial; IUI, intrauterine insemination; P, progesterone; RIF, recurrent implantation failure; ET, embryo transfer.

The first reported use of intrauterine PRP infusion to enhance EMT in women with thin endometrium undergoing IVF was published by Chang et al., in 2015. In this study, five patients with a history of cycle cancelation due to thin endometrium ( ≤ 7 mm) under conventional hormone replacement therapy (HRT) were recruited. PRP was infused into the uterine cavity on the 10th day of the HRT cycle, with a repeat infusion option if EMT remained unsatisfactory after 72 h. The results showed a significant increase in EMT, with all patients achieving >7 mm by the day of progesterone administration. All five patients achieved pregnancy, although one resulted in a missed abortion due to chromosome abnormalities (Chang et al., 2015).

### 2.1 Cohort studies

Subsequent case series and prospective studies have further supported the efficacy of intrauterine PRP infusion, demonstrating both improvements in EMT and favorable implantation rates (Table 1). A case series involving 10 patients, aged 30 to 39, with a history of frozen embryo transfer (FET) cycle cancellations due to refractory thin EMT (<7 mm), showed promising results. Uterine cavity abnormalities were ruled out through hysteroscopy prior to initiating treatment. PRP infusions were administered on days 11–12 and 13–14 of the HRT days. Following two PRP doses, all patients achieved an EMT greater than 7 mm. Five patients had positive pregnancy tests, with four progressing to clinical pregnancies (Zadehmodarres et al., 2017). A prospective study further reinforced these findings, reporting significant increases in EMT and an implantation rate of 73.7% after two PRP infusions. This study included 19 patients aged 33 to 45, all with a history of refractory thin endometrium and at least one prior failed IVF attempt. PRP infusions were given on days 10 and 12 of the HRT cycle. Following the first infusion, all patients reached an EMT of ≥7 mm, and following the second infusion, the mean EMT increased to 9 mm. Fourteen out of 19 patients (73.7%) had positive pregnancy tests; however, only 5 (26.3%) resulted in ongoing pregnancies and live births (Molina et al., 2018).

While most studies have focused on PRP’s role in endometrial thickening, some have evaluated its effect on endometrial receptivity. One case series enrolled 21 patients with high-quality day 5/day six embryos (graded greater than over equal to 3BB) but with a persistent thin endometrium (<5 mm) by days 14–17 of their HRT cycle. PRP infusions were administered every other day for a total of three treatments. Among 24 completed cycles, the clinical pregnancy rate was 66.7% (16/24), and the ongoing pregnancy/live birth rate was 54% (13/24). These findings suggested that endometrial receptivity, rather than EMT alone, may play a crucial role in pregnancy success (Frantz et al., 2020).

A study by Kim et al. further challenged the notion that increased EMT is the primary factor driving improved pregnancy outcomes. This study included 24 patients with a history of at least two failed IVF cycles and refractory thin endometrium. Notably, 16 of these patients had hysteroscopic evidence of endometrial adhesion or sclerosis. PRP infusions were administered on day 10 of the HRT cycle and repeated every 3 days for up to three treatments until the EMT reached ≥7 mm. Despite a modest mean EMT increase (from 5.4 mm to 6.0 mm, p = NS), significant improvements were observed in implantation (12.7%), clinical pregnancy (30%), and live birth rates (20%). Interestingly, in four out of six clinical pregnancies, EMT actually decreased post-PRP infusion, further supporting the hypothesis that PRP enhances endometrial receptivity through mechanisms beyond endometrial thickening (Kim et al., 2019).

Lin et al. investigated factors associated with PRP treatment failure in endometrial expansion and pregnancy outcomes. The study enrolled 77 women with recurrent implantation failure (RIF) (defined as 4 cumulative transfers of good-quality cleavage-stage embryos or blastocysts) and EMT <7 mm on HRT cycle days 11–13. Patients received two intrauterine PRP infusions at an interval of 48 h apart, and their EMT reassessed 3 days later. Among them, 11 patients (14.3%) had ET canceled due to persistent thin endometrium (<7 mm). The remaining 66 patients proceeded with ET under optimal EMT (>7 mm). The cancel group had significantly lower baseline EMT before PRP infusion (5.7 ± 1.3 vs 6.4 ± 0.7, p = 0.02). In the ET group, EMT increased significantly, from 6.4 mm to 8.3 mm, with implantation, clinical pregnancy, and live birth rates of 21%, 33%, and 21%, respectively. Multivariate analysis identified baseline EMT as the sole predictor of endometrial expansion following PRP treatment, whereas a higher number of prior uterine surgeries was negatively correlated with clinical pregnancy rates, underscoring the detrimental effects of repeated uterine injuries on reproductive outcomes (Lin et al., 2023).

In 2022, Dogra et al. explored PRP infusion in women undergoing fresh embryo transfer. This prospective cohort study included 20 women under 38 years old who with a history of FET cycle cancellation due to persistent thin endometrium (<7 mm) despite standard HRT. The participants were divided into two groups: those preparing for FET cycles (12 cycles) and those undergoing fresh IVF cycles (14 cycles). In the FET group, PRP infusion was administered on day 8 of HRT if EMT remained <7 mm, with repeat doses every 48 h as needed. In the fresh cycle group, PRP infusion began on day 8 of ovarian stimulation. Significant EMT expansion was observed in both fresh (5.83 ± 0.81 mm vs 7.14 ± 0.54 mm, p < 0.001) and FET cycles (5.52 ± 0.89 mm vs 7.14 ± 0.68 mm, p < 0.001), with no significant differences between the two groups. The live birth rate per transfer was 33.3% (4/12) for fresh cycles and 9.1% (1/11) for FET cycle, marking the first study to report live birth outcomes after PRP infusion in non-donor fresh IVF cycles (Dogra et al., 2022).

### 2.2 Comparative studies

To date, four comparative studies have investigated the efficacy of intrauterine PRP infusion for women with thin endometrium undergoing IVF treatment (Table 1).

The first randomized control trial (RCT), conducted by Eftekhar et al., in 2018, enrolled 83 patients with poor endometrial response (EMT <7 mm) on the 13th day of HRT. Participants were randomly assigned to either the PRP group, which received intrauterine PRP infusion alongside estradiol (10 mg/day), or the control group, which continued conventional HRT with the same estradiol dosage. Progesterone was initiated once EMT reached 7 mm; otherwise, the cycle was canceled by day 17. Although the cycle cancellation rate was lower in the PRP group (17.5%, 7/40) compared to the control group (23.2%, 10/43), the difference was not statistically significant. However, among patients who proceeded to embryo transfer, the PRP group demonstrated significantly greater EMT (8.67 ± 0.64 vs 8.04 ± 0.27 mm, p = 0.001) and a higher clinical pregnancy rate per cycle (32.5% vs 14%, p = 0.044). Ongoing pregnancy rates were comparable between the groups (Eftekhar et al., 2018).

The second RCT, a double-blind study published in 2019, examined 60 patients under 38 years old with a history of FET cycle cancellations due to refractory thin endometrium (<7 mm). Participants were randomly assigned to either the PRP group (n = 30), which received intrauterine PRP infusion *via* an intrauterine insemination (IUI) catheter, or the control group (n = 30), which underwent a sham catheter procedure. PRP infusions were administered on days 11–12 of the HRT cycle, and a second infusion was performed 48 h later if EMT remained inadequate. While no significant difference in EMT was observed after the first infusion, the second infusion resulted in significantly greater EMT in the PRP group compared to controls (7.21 ± 0.18 vs 5.76 ± 0.97 mm, p < 0.001). In the control group, 24 patients (80%) had their cycles canceled due to persistent thin endometrium, whereas all 30 patients in the PRP group proceeded to embryo transfer with adequate endometrial growth. The clinical pregnancy rate was significantly higher in the PRP group (33.3%, 10/30), compared to the control group (3.3%, 1/30) (Nazari et al., 2019).

A non-randomized controlled trial further evaluated the effects of PRP infusion in 64 patients with a history of cycle cancellation due to refractory thin endometrium. All patients underwent HRT cycles and were allocated to either the PRP group (n = 34) or the control group (n = 30) based on patient preference. PRP infusions were administered on the 10th day of the HRT cycle and again on the day of progesterone initiation. Compared to the control group, the PRP group demonstrated significantly greater EMT (7.65 ± 0.22 vs 6.52 ± 0.31 mm, p = 0.013), as well as higher implantation (27.94% vs 11.67%, p = 0.018) and clinical pregnancy rates (44.12% vs 20%, p = 0.036). Despite 8 out of 34 patients in the PRP group requiring cycle cancellation due to persistent thin endometrium, the cycle cancellation rate remained significantly lower in the PRP group compared to controls (19.05% vs 41.18%) (Chang et al., 2019).

A prospective, single-arm controlled study investigated the impact of PRP infusion in 36 patients with repeated implantation failure (mean of 5.6 failed cycles) and refractory thin endometrium (<7 mm). The majority (n = 30) had a history of gynecological surgery, predominantly involving the endometrium. Patients underwent two consecutive HRT cycles, with PRP infusions administered on the 10th and 12th days of the second cycle. EMT measurements were taken on days 10 and 14 of both cycles using blind and unblinded assessments. Compared to the first cycle (HRT alone), the EMT on day 14 was significantly thicker in the second cycle (HRT + PRP) based on both unblinded (7.25 mm vs 5.98 mm, p < 0.001) and blinded (6.76 mm vs 6.04 mm, p < 0.001) evaluations. A total of 32 patients proceeded with FET at a mean EMT of 7.13 mm. The implantation rate (defined as the presence of a gestational sac) was 13.9% (5/36 per transferred embryo), and the clinical pregnancy rate was 15.6% (5/32). Interestingly, three patients conceived despite having an EMT <7 mm at transfer, suggesting that PRP may enhance endometrial quality and receptivity beyond its effects on endometrial thickening (Kusumi et al., 2020).

These studies collectively underscore the potential benefits of intrauterine PRP infusion in improving EMT, reducing cycle cancellation rates, and enhancing clinical pregnancy outcomes in women with refractory thin endometrium. Although findings are promising, further large-scale randomized controlled trials are needed to confirm the long-term benefits and establish standardized treatment protocols for PRP application in assisted reproductive technology.

### 2.3 Sub-endometrial injection of PRP

In 2020, Agarwal et al. introduced a novel approach for PRP administration *via* sub-endometrial injection guided by hysteroscopy. This retrospective study included 32 women aged 27 to 39 with a history of FET cancellation due to persistent thin endometrium (<7 mm) despite increased estrogen dosages. The cohort also included patients who had previously undergone intrauterine PRP infusion (n = 7) or granulocyte-colony stimulating factor G-CSF treatment (n = 13). To prepare for the procedure, patients were administered oral contraceptive pills (OCPs) and leuprolide in the preceding cycle. Hysteroscopic-guided PRP injections were performed 7–10 days after leuprolide administration, with 4 mL of autologous PRP injected into the four walls of the uterine cavity using an ovum pickup needle. The procedure was well tolerated, with no adverse effects reported. Following treatment, 24 out of 32 patients (75%) achieved an EMT of ≥7 mm, while 8 (25%) failed to do so and had their cycles canceled. Among those who proceeded to FET, 10 achieved clinical pregnancy (41.66%), with 5 resulting in live births (20.83%) (Agarwal et al., 2020).

In 2024, Yu et al. conducted a prospective case-control study comparing the efficacy of intrauterine infusion *versus* hysteroscopic injection of PRP in women with thin endometrium undergoing euploid FET (EFET). The study included 116 infertile women with a history of at least one failed EFET due to refractory thin endometrium. On days 11–13 of the HRT cycle, endometrial thickness was measured *via* transvaginal ultrasound. Patients with an EMT <7 mm were given the choice to undergo intrauterine PRP infusion, hysteroscopic PRP injection, or to continue HRT alone (control group). Ultimately, 55 patients opted for intrauterine infusion, 38 for hysteroscopic injection, and 23 remained in the control group. In the intrauterine infusion group, 2 mL of autologous PRP was infused into the uterine cavity *via* an embryo transfer catheter, with a second infusion administered 48 h later if necessary. In the hysteroscopic injection group, PRP (2 mL total) was injected into the endometrium at a depth of 2–3 mm in four directions (upper, lower, right, and left walls of the uterine cavity), with 0.5 mL administered at each site. Endometrial thickness was reassessed 2–3 days post-treatment. Both PRP treatment groups demonstrated significant EMT expansion compared to pre-treatment measurements. Post-treatment EMT was 8.2 ± 1.8 mm in the intrauterine infusion group and 7.5 ± 1.8 mm in the hysteroscopic injection group, both significantly greater than pre-treatment EMT and significantly thicker than in the control group on the day of embryo transfer (p < 0.001). EMT ≥7 mm was achieved in 78.2% (43/55) of the intrauterine infusion group and in 55.3% (21/38) of the hysteroscopic injection group. Interestingly, despite the greater increase in EMT observed with intrauterine infusion, the hysteroscopic injection group exhibited superior pregnancy outcomes. The implantation rate (52% vs 18%, p < 0.001) and live birth rate (38% vs 4%, p < 0.001) were significantly higher in the hysteroscopic injection group compared to the control group. In contrast, while the intrauterine infusion group showed higher implantation (27% vs 18%) and clinical pregnancy rates (33% vs 22%) compared to controls, these differences were not statistically significant (p > 0.05). These findings suggest that while intrauterine infusion is more effective in EMT expansion, hysteroscopic PRP injection may have a greater impact on endometrial receptivity and implantation potential (Yu et al., 2024).

Sub-endometrial PRP injection is an emerging therapeutic option for patients with refractory thin endometrium, demonstrating both safety and efficacy in improving endometrial thickness and implantation potential (Table 2). While intrauterine PRP infusion enhances EMT expansion, hysteroscopic PRP injections appear to confer superior pregnancy outcomes, possibly due to its targeted delivery and localized effects on endometrial receptivity (Table 2). Further large-scale clinical trials and mechanistic studies are warranted to establish standardized treatment protocols and maximize the benefits of PRP in reproductive medicine.

**TABLE 2:** G-CSF therapy for refractory thin endometrium (case series and cohort study).

Study	Objective	Samples	ET protocol	G-CSF dosage [Timing]	EMT before G-CSF (mm)	EMT after G-CSF (mm)	EMT difference	Pregnancy outcomes
Gleicher et al. (2011) Case series	EMT <7 mm days before ET	4	FET	300 mcg/1 mL	—	—	All reach 7 mm in 2 days	• All pregnancy (1 intramural pregnancy)
Gleicher et al. (2013) Prospective, cohort	EMT <7 mm on hCG day	21	Fresh ET	300 mcg/1 mL [1st: 6–12 h before hCG] [2nd: after OPU]^a^	6.4 ± 1.4	9.3 ± 2.1	Significant increase (p < 0.001)	• CPR: 4/21, 19.1%
Kunicki et al. (2014) Prospective cohort	History of cycle cancellation due to thin EM; EMT <7 mm on hCG day	37	Fresh ET	300 mcg/1 mL [hCG day]	6.74 ± 1.75	8.42 ± 1.73	Significantly increased 3 days after G-CSF (p < 0.001)	• CPR: 7/37, 18.9%
Shah et al. (2014) Cohort	Subgroup 1 Persistent EMT <8 mm despite HRT	117	FET (HRT)	300 mcg [After D10 of HRT]	Mean increment: 3.24 ± 0.86		111/117 (95%) increase EMT 4 days after G-CSF	• Positive hCG: 56/111, 50.45% • CPR: 41/111, 37%
Swati et al. (2015) Prospective cohort	EMT <7 mm on the D20 of HRT	250	FET	NM	NM		No significant increment (p = 0.09) 73/220 (33.18%) reach EMT >7 mm 2 days after G-CSF	• CPR: 25/73, 34.2%
Tehraninejad et al. (2015) Cohort	History of cycle cancellation due to EMT <6 mm	15	Fresh ET	300 mcg/1 mL [OPU day]	3.6 ± 0.98	7.12 ± 0.84	Significantly increased 5 days after G-CSF (p < 0.001)	• CPR: 3/13, 23.1%
Lee et al. (2016) Retrospective	EMT ≤8 mm on hCG day during the previous cycle	50	Fresh ET	300 mcg/1 mL n = 12 [hCG day] n = 38 [After OPU]	7.2 ± 0.6 (hCG day)	8.5 ± 1.5 (ET day)	Significant increase (p < 0.001)	• CPR: 11/50, 22%
Mishra et al. (2016) Prospective cohort	History of cycle cancellations due to thin EM; EMT <7 mm on D14 of HRT	35	FET (HRT)	300 mcg/1 mL [1st: D14 of HRT] [2nd: D16 of HRT]	5.86 ± 0.58	6.58 ± 0.84	A significant increase (p < 0.01), but mean EMT after G-CSF did not reach 7 mm	• Cycle cancellation: 16/35, 45.7% • Chemical pregnancy: 3/19, 15.78% • CPR: 0/19, 0%

#### 2.3.1 Meta-analysis

Currently, due to the limited number of comparative studies evaluating intrauterine PRP infusion for patients with persistent thin endometrium undergoing IVF, no comprehensive large-scale meta-analysis has been conducted in the area. A 2020 meta-analysis included seven studies investigating intrauterine PRP infusion. However, the majority focused on women with RIF, with only two studies specifically addressing patients with refractory thin endometrium. An analysis of these two studies, comprising 74 intervention subjects and 73 controls, demonstrated a significant increase in EMT among those receiving PRP compared to the control group (Maleki-Hajiagha et al., 2020). Similarly, a 2024 Cochrane review included nine studies on intrauterine PRP injection, yet most focused on RIF rather than patients with refractory thin endometrium (Vaidakis et al., 2024). The limited number of studies targeting this specific population highlights the need for further well-designed, randomized controlled trials to better elucidate PRP’s role in optimizing endometrial conditions and improving reproductive outcomes (Table 2).

In summary, PRP therapy has shown promise in improving endometrial thickness and enhancing implantation rates in women with refractory thin endometrium undergoing assisted reproductive treatments. Studies have demonstrated that intrauterine PRP infusion leads to significant EMT expansion and increased pregnancy rates, while hysteroscopic sub-endometrial PRP injections may further enhance endometrial receptivity and implantation success (Table 2). Despite encouraging results, a standardized PRP preparation and administration protocol remains to be established, warranting further large-scale clinical trials.

## 3 Granulocyte colony-stimulating factor (G-CSF) therapy

G-CSF is a glycoprotein with both growth factor and cytokine functions (Gleicher et al., 2013), synthesized by various cells, including bone marrow stromal cells, mononuclear cells, fibroblasts, and endometrial cells (Fu et al., 2023). Initially used to prevent neutropenia by stimulating the proliferation and differentiation of neutrophilic granulocytes, G-CSF also plays a role in reproductive processes such as follicular development, oocyte maturation, blastocyst formation, and implantation. Although its direct effects on endometrium remain unclear, its potential mechanism may involve immunological interactions among glandular epithelial cells, stromal cells, and immune cells within the endometrium (Xie et al., 2017).

In 2011, Gleicher et al. investigated the efficacy of intrauterine G-CSF perfusion in infertile women with persistent thin endometrium. The case series included 4 participants with inadequate EMT (<7 mm) in the days preceding ET, despite treatment with estrogen and vaginal sildenafil. The patients, aged 33–45 years, included three undergoing egg donor cycles. Each received an intrauterine infusion of G-CSF (300 μg/mL), resulting in a rapid EMT increase to at least 7 mm within 48 h. Remarkably, all patients conceived, although one experienced a cornual pregnancy, suggesting that G-CSF may enhance endometrial receptivity in cases of refractory thin endometrium (Gleicher et al., 2011).

### 3.1 Cohort studies on fresh embryo transfer

Two years later, the same author reported a pilot study involving 21 participants who received intrauterine G-CSF under fresh ET cycles. All participants had thin endometrium (<7 mm) on the day of the ovarian trigger, despite prior treatment with estrogen and vaginal sildenafil. G-CSF was administered by intrauterine infusion 6–12 h before the human chorionic gonadotropin (hCG) trigger. Endometrial thickness (EMT) was reassessed during oocyte retrieval, and if it remained below 7 mm, a second infusion was performed. Only three participants required a second infusion. The results demonstrated a significant increase in EMT from the first G-CSF infusion to ET day (6.4 ± 1.4 mm vs 9.3 ± 2.1 mm, p < 0.001). Ultimately, all participants achieved an EMT of at least 7 mm and proceeded with fresh ET, resulting in a clinical pregnancy rate of 19.1% (4/21). The study highlighted the rapid endometrial expansion within 48 h of G-CSF administration (Gleicher et al., 2013).

Similar studies were later conducted. Kunicki et al. recruited 37 patients with a history of cycle cancellation due to refractory thin endometrium (<7 mm) and inadequate EMT on hCG trigger day. Following intrauterine G-CSF infusion at the time of hCG administration, using the same protocol, a significant EMT increase was observed within 72 h (6.74 ± 1.75 mm vs 8.42 ± 1.73 mm, p < 0.001). While EMT expansion was greater in patients who conceived (1.94 ± 0.99 mm) compared to those who did not (1.62 ± 1.07 mm), the difference was not significant (p = 0.848). All patients underwent fresh ET, with a clinical pregnancy rate of 18.9% (7/37), closely mirroring the findings of Kunicki et al. (2014). Tehraninejad et al. conducted a similar study on 15 patients with even thinner EMT (<6 mm), administering G-CSF on the day of oocyte retrieval at the same dosage. Within 5 days, EMT significantly increased from 3.6 ± 0.98 mm to 7.12 ± 0.84 mm. Twelve out of 15 patients proceeded with fresh ET, achieving a slightly higher clinical pregnancy rate (3/13, 23.1%) (Tehraninejad et al., 2015).

Additionally, Lee et al. performed a pilot study evaluating the timing of G-CSF infusion, comparing administration on the ovulation trigger day *versus* oocyte retrieval day. A significant overall EMT increase was observed (7.2 ± 0.6 to 8.5 ± 1.5 mm, p < 0.001), with a notably greater improvement when G-CSF was infused on the ovulation trigger day (Lee et al., 2016). Overall, these studies suggest that G-CSF infusion effectively enhances EMT in fresh ET cycles, with promising pregnancy outcomes.

### 3.2 Cohort studies on frozen embryo transfer

In 2014, Shah et al. conducted a prospective study involving 231 patients undergoing FET cycle. These patients were divided into two subgroups: Subgroup I (thin endometrium, n = 117), included patients with an EMT <8 mm despite estradiol valerate and vaginal sildenafil priming, while Subgroup II (recurrent implantation failure or RIF, n = 114) included patients with at least two implantation failures despite an adequate EMT (≥8 mm). All patients received an intrauterine G-CSF infusion (300 μg) after a 10-day endometrial priming period, followed by EMT assessment 4 days later, before initiating progesterone. In Subgroup I, G-CSF infusion resulted in a significant EMT increase of 3.24 ± 0.86 mm (p < 0.0001), with a greater response observed in this group compared to Subgroup II. Among the 111 patients in Subgroup I, 56 had a positive pregnancy test (50.45%), and the clinical pregnancy rate was 37% (41/111) (Shah et al., 2014).

A larger study by Swati et al., which included 250 women with thin endometrium, found no significant improvement in EMT following G-CSF infusion. Among the participants, only one-third achieved an EMT above 7 mm within 48 h after treatment, suggesting a limited response in the majority of cases. Despite the lack of a substantial increase in EMT, the clinical pregnancy rate remained comparable to Shah et al.’s findings at 34.2% (Swati et al., 2015). This indicates that while G-CSF may not consistently enhance endometrial thickness, it could still contribute to endometrial receptivity or other implantation-related mechanisms. The study highlights the variability in patient response to G-CSF therapy and underscores the need for further research to identify predictive factors for treatment success, optimal patient selection, and potential combination strategies with other endometrial priming protocols.

Similarly, Mishra et al. found no benefits of G-CSF on clinical pregnancy rates in patients with thin refractory endometrium. In their study of 35women with previous cycle cancellations due to thin EMT (<7 mm), participants received intrauterine G-CSF (300 μg/1 mL) after 14 days of endometrial priming with estradiol valerate, aspirin, and vaginal sildenafil. If EMT remained less than 7 mm after 48 h, a second infusion was administered, or the cycle was terminated. Despite a significant increase in EMT post-G-CSF (5.86 ± 0.58 mm vs 6.58 ± 0.84 mm, p < 0.01), the mean EMT remained below 7 mm. Additionally, G-CSF did not affect endometrial volume or vascular indices. Among the 35 participants, 19 achieved an EMT ≥7 mm and proceeded with FET, but only three experienced biochemical pregnancies (3/19, 15.78%), and none achieved a clinical pregnancy (0/19, 0%) clinical pregnancy (0/19, 0%) (Mishra et al., 2016).

These findings suggest that while G-CSF may improve EMT in some cases, its impact on pregnancy outcomes in FET cycles remains inconsistent, warranting further investigation (Table 2). The variability in response across studies highlights the need to better understand the mechanisms by which G-CSF influences endometrial receptivity and implantation. Additionally, differences in study design, patient populations, and timing of G-CSF administration may contribute to the inconsistent outcomes observed. Future research should focus on identifying biomarkers that predict response to G-CSF therapy, optimizing dosage and administration protocols, and exploring potential synergistic effects with other endometrial priming strategies.

### 3.3 Non-randomized controlled trials

Several non-randomized controlled trials have investigated the effects of G-CSF in FET cycles, with most failing to demonstrate significant benefits for endometrial expansion or pregnancy outcomes (Table 3).

**TABLE 3:** G-CSF therapy in thin endometrium (controlled trial).

Study	Objective	Samples [Cycles]	ET protocol	G-CSF dosage [Timing]	Control group EMT (mm)	G-CSF group EMT (mm)	EMT difference	Pregnancy outcomes (G-CSF vs control)
Li et al. (2014) Retrospective, non-randomized control	EMT <7 mm on ovulation/hCG/P start day	G-CSF: 34 (Mishra et al., 2016) Self-control: 34 (Li et al., 2017) Control: 25 (Zhang et al., 2022)	FET (NC/induced/HRT cycle)	100 mcg/0.6 mL [Ovulation day] or [hCG day] or [P start day]	G-CSF group		No significant difference after G-CSF (p = 0.403)	• No difference in IR and CPR among 3 groups (p > 0.05) G-CSF vs self-control vs control IR: 15.85% vs 7.89% vs 13.33% CPR: 30.3% vs. 20% vs 29.27%
					6.53 ± 0.65 (Ovulation day)	6.75 ± 1.17 (ET day)		
Eftekhar et al. (2014) Non-randomized control	EMT <7 mm on D12-13 of HRT	G-CSF: 34 Control: 34	FET (HRT)	300 mcg [1st: D12-13 of HRT] [2nd: 48–72 h later]^a^	8.23 ± 0.8 (P start day)	7.91 ± 0.55 (P start day)	No significant difference (p = 0.1)	No difference in CPR: 32.1% vs 12.0% (p = 0.1)
Szlarb et al. (2014) Non-randomized control	EMT <7 mm; oocyte recipients	G-CSF: 29 Control: 18	FET	34 IU [2–3 days before ET]	6.5 ± 1.7 (ET day)	7.3 ± 1.1 (ET day)	No significant difference	• No difference in CPR: 34.4% vs 11.1% (p = 0.08)
Xu et al. (2015) Case control	EMT <7 mm on ovulation/hCG/P start day	G-CSF: 30 (G-CSF-only: 15; G-CSF + EMS: 15) Control: 52	FET (NC/induced)	300 mcg/1.8 mL [The day that one follicle became dominant]	G-CSF only group		Significantly thicker after G-CSF (p = 0.003)	• Significantly higher IR and CPR IR: 31.5% vs 13.9% (p < 0.01) CPR: 48.1% vs 25.0% (p = 0.0038) • No difference in LBR: 33.3% vs 17.3%
					5.7 ± 0.7 (before G-CSF)	8.1 ± 2.5 (after G-CSF)		
Singh et al. (2015) RCT	Thin endometrium	G-CSF: 24 Control: 24	Fresh ET	300 mg/mL [1st: hCG day] [2nd: after OPU]^a^	G-CSF group		Significantly thicker after G-CSF and in the G-CSF group	• Significantly higher IR and CPR IR: 23% vs 12% CPR: 33% vs 16%
					6.49 ± 1.65	8.79 ± 1.57		
Singh and Singh (2018) RCT	Thin endometrium	G-CSF: 56 Control: 56	Fresh ET	300 mcg/1 m (Intrauterine infusion/SC) [1st: hCG day] [2nd: after OPU]^a^	G-CSF group		Significantly thicker after G-CSF and in the G-CSF group	• Significantly higher IR and CPR IR: 24% vs 11% • CPR: 34% vs 16%
					6.23 ± 1.45	8.46 ± 1.27		
Sarvi et al. (2017) RCT	History of thin EM; EMT <6 mm on hCG day	G-CSF: 13 Control:15	Fresh ET	300 mcg/1 mL [1st: hCG day] [2nd: ET day]^b^	6.3 ± 1 (OPU day) 6.9 ± 1.1 (ET day)	8 ± 1 (OPU day) 9.1 ± 1.5 (ET day)	Significantly thicker in the G-CSF group (p = 0.001)	• Significant higher IR: 10.3% vs 5.4% (p < 0.001) • No difference in CPR: 15.3% vs 20% (p = 0.7)
Lian et al. (2020) Retrospective, non-randomized control	History of cycle cancellations due to thin EM EMT <7 mm on P start day	G-CSF: 117 Control: 154	FET (HRT)	100 mcg/0.4 mL [1st: D8 of HRT] [2nd: D11 of HRT] [3rd: D14 of HRT]	G-CSF group		Significantly thicker after G-CSF (p < 0.001)	• No difference between IR and CPR IR: 27.52% vs. 32.08% (p = 0.267) CPR: 41.03% vs. 48.70% (p = 0.264)
					6.13 ± 0.94 (before G-CSF)	6.91 ± 1.17 (after G-CSF)		

Abbreviations: IVF, *in vitro* fertilization; G-CSF, granulocyte colony-stimulating factor; EMT, endometrial thickness; FET, frozen embryo transfer; ET, embryo transfer; hCG, human chorionic gonadotropin; OPU, oocyte pick-up; CPR, clinical pregnancy rate; EM, endometrium; HRT, hormone replacement therapy; NM, not mentioned; P, progesterone; NC, natural cycle; IR, implantation rate; LBR, live birth rate; SC, subcutaneously.

^a^ If EMT <7 mm.

^b^ If EMT <6 mm.

One such study by Li et al., in 2014 assessed 59 women with thin endometrium (<7 mm) undergoing FET under natural, extended estrogen, or induced ovulation cycle. Patients with EMT <7 mm on ovulation day or progesterone administration day were offered G-CSF treatment. Of these, 34 patients received intrauterine G-CSF infusion (100 µg/0.6 mL), forming the G-CSF group (40 cycles), defined as G-CSF group, while their previous FET cycles without G-CSF (49 cycles) served as a self-controlled group. An additional 25 patients (80 cycles) who refused G-CSF formed the control group. Although the G-CSF group showed a higher implantation rate (15.85% vs 7.89%, p > 0.05) and clinical pregnancy rate (30.3% vs 20%, p > 0.05) compared to the self-controlled group, the differences were not statistically significant. Moreover, EMT before and after G-CSF infusion remained similar (6.53 ± 0.65 mm vs 6.75 ± 1.17 mm, p = 0.403). The authors suggested that the poor endometrial condition of participants and the lower G-CSF dose (100 µg) compared to Gleicher et al.’s study (300 µg) may have influenced the results (Li et al., 2014).

Eftekhar et al. also failed to demonstrate the efficacy of G-CSF in improving endometrial thickness or pregnancy outcomes. In their study, 68 women with EMT <7 mm after 12–13 days of HRT were allocated to either a G-CSF group (n = 34) or a control group (n = 34). Patients in the G-CSF group received an intrauterine infusion of 300 μg G-CSF, while the control group continued standard estrogen treatment. If EMT remained <7 mm, a second infusion (G-CSF group) or continued estradiol (control group) was provided. Six patients in the G-CSF group required a second infusion. Ultimately, the EMT on the first progesterone day was comparable between the groups (7.91 ± 0.55 mm vs 8.23 ± 0.82 mm, p = 0.10). Although the G-CSF group exhibited higher chemical (39.3% vs 14.3%, p = 0.68) and clinical pregnancy rates (32.1% vs 12%, p = 0.10), the differences were not statistically significant, likely due to the small sample size (Eftekhar et al., 2014). Another study involving 47 women undergoing an oocyte donation program infused 34 IU of G-CSF 2–3 days before ET. No significant improvement in EMT on ET day or clinical pregnancy rate (34.4% vs 11.1%, p = 0.08) was observed (Szlarb et al., 2014). Lian et al. (2020) conducted a retrospective study on 271 women with a thin endometrium (<7 mm) under HRT-FET cycles. Among them, 117 received intrauterine G-CSF (100 µg/0.4 mL) on days 8, 11, and 14 of the cycle, while 154 served as controls. Although EMT significantly increased post-G-CSF (6.13 ± 0.94 mm to 6.91 ± 1.17 mm, p < 0.001), implantation (27.52% vs 32.08%, p = 0.267) and clinical pregnancy rates (41.03% vs 48.70%, p = 0.264) remained similar between groups. Further analysis within the G-CSF group revealed a greater, but statistically insignificant, EMT increase in those who conceived compared to those who did not (0.96 ± 1.22 vs 0.66 ± 1.09 mm, p = 0.169) (Lian et al., 2020).

In contrast, Xu et al. (2015) reported promising results. They studied 82 women with thin endometrium during fresh or FET cycles and offered them two options: canceling the cycle and attempting FET with G-CSF in a subsequent cycle or proceeding with ET despite inadequate EMT. Ultimately, 30 patients received G-CSF (300 µg/1.8 mL) under natural or induced ovulation FET cycles. Patients were randomly assigned to “G-CSF-only” or “G-CSF + endometrial scratch (EMS)” subgroups. Intrauterine G-CSF was infused when a dominant follicle (≥12 mm) emerged. EMT was measured on days 2, 4, 5, and 6 post-infusions. Three patients in the G-CSF group canceled ET due to poor EMT. The G-CSF group had significantly higher implantation (31.5% vs 13.9%, p < 0.01) and clinical pregnancy rates (48.1% vs 25.0%, p = 0.038) compared to controls, though live birth rates were similar (33.3% vs 17.3%). EMT significantly increased in the G-CSF-only group from 5.7 ± 0.7 mm to 8.1 ± 2.5 mm (p = 0.003). Both subgroups demonstrated EMT expansion, though no significant difference between them was noted. However, limitations included: (1) three patients in the G-CSF group were excluded due to poor EMT and (2) a lack of direct comparison between the G-CSF-only group and controls, preventing conclusions about the isolated effect of G-CSF (Xu et al., 2015).

Overall, these non-randomized trials present conflicting findings, with most studies failing to demonstrate a significant benefit of G-CSF in improving pregnancy outcomes, despite occasional increases in EMT. While some studies reported a modest rise in EMT following G-CSF infusion, this did not consistently translate into higher implantation, clinical pregnancy, or live birth rates. The variability in study designs, patient populations, treatment protocols, and G-CSF dosages further complicates the interpretation of results. Additionally, small sample sizes and the absence of proper randomization in most studies limit their reliability and generalizability.

### 3.4 Randomized controlled trials

Singh et al. conducted multiple studies assessing the efficacy of G-CSF treatment for women with thin endometrium undergoing IVF cycles. In a RCT published in 2015, 48 women with thin endometrium were randomly assigned to either the G-CSF group or the control group. Participants in the G-CSF group received an intrauterine infusion of G-CSF on the day of oocyte trigger. If the EMT remained below 7 mm on the day of oocyte retrieval, a second infusion was administered. In contrast, women in the control group received a saline infusion as a placebo. The results showed a significantly greater EMT in the G-CSF group compared to the control group. Pregnancy outcomes were also more favorable, with a significantly higher implantation rate (23% vs 12%) and clinical pregnancy rate (33% vs 16%) in the G-CSF group (Singh et al., 2015). In a subsequent RCT published in 2018, Singh et al. increased the sample size, enrolling 56 women in each group. This study introduced an additional variation in the treatment protocol, as women in the G-CSF group received either intrauterine G-CSF infusion or subcutaneous G-CSF injection. The results remained promising, demonstrating significant improvements in both EMT and pregnancy outcomes, further supporting the potential benefits of G-CSF (Singh and Singh, 2018).

Similarly, Sarvi et al. (2017) investigated the effect of G-CSF on EMT expansion and implantation rates in women undergoing fresh embryo transfer. The study included 34 women with at least one previous IVF cycle characterized by persistent thin endometrium despite treatment. All participants had a normal uterine cavity confirmed by hysteroscopy and no history of uterine surgery. Patients underwent ovarian stimulation using the long protocol, and those with an EMT of less than 6 mm on the day of hCG administration were randomly assigned to either the G-CSF group (n = 13) or the control group (n = 15). In the G-CSF group, a 300 μg/mL dose of G-CSF was infused into the uterine cavity, whereas the control group received a 1 mL normal saline infusion. EMT was assessed on the day of oocyte retrieval, and if it remained below 6 mm, a second dose of G-CSF was administered 2–3 days post-retrieval. The embryo transfer was performed on the same day afterward. Three women in the G-CSF group received this second dose, while none in the control group did. The study findings demonstrated a significantly thicker EMT in the G-CSF group compared to the control group, both on the day of oocyte retrieval (8 ± 1 mm vs 6.3 ± 1 mm, p = 0.001) and the day of embryo transfer (9.1 ± 1.5 mm vs 6.9 ± 1.1 mm, p = 0.001). The implantation rate was significantly higher in the G-CSF group (10.3% vs 5.4%, p < 0.001). However, no significant difference was observed in clinical pregnancy rates between the two groups (15.3% vs 20%, p = 0.7) (Sarvi et al., 2017). These findings suggest that while G-CSF effectively increases EMT and implantation rates, its impact on sustained pregnancy outcomes remains uncertain.

Overall, these RCTs provide encouraging evidence that G-CSF may enhance EMT and implantation rates in women with thin endometrium (Table 3). However, the variability in treatment protocols, small sample sizes, and lack of consistent improvement in clinical pregnancy rates highlight the need for larger, well-designed trials to confirm its efficacy and determine the optimal route, dosage, and timing of administration.

### 3.5 Meta-analysis

In 2017, Li et al. published a meta-analysis examining the effect of G-CSF on infertile women undergoing IVF treatment. Due to the high heterogenicity among studies, the authors performed a subgroup analysis, categorizing participants into two groups: the “thin endometrium or RIF” subgroup and the “normal IVF” subgroup. In the “thin endometrium or RIF” group, three studies focused on women with thin endometrium—Li et al. (2014), Eftekhar et al. (2014) and Xu et al. (2015) —while one study focused on RIF. The final pooled analysis demonstrated significant improvements in implantation rate (Risk ratio, RR = 1.887, 95% confidence interval, CI: 1.256–2.833), biochemical pregnancy rate (RR = 2.385, 95% CI: 1.414–4.023), and clinical pregnancy rate (RR = 2.312, 95% CI: 1.444–3.701) following G-CSF infusion in the “thin endometrium or RIF” subgroup. However, no significant difference in EMT was observed, probably due to the diverse patient populations included in the subgroup. Overall, the findings suggest that G-CSF may enhance pregnancy outcomes in women with thin endometrium or repeated IVF failures (Li et al., 2017).

In the same year, Xie et al. published another systematic review and meta-analysis, specifically focused on infertile women with thin endometrium. This study included 11 articles: one RCT, four non-randomized controlled studies, and six cohort studies. Notably, in the cohort studies, control group data were obtained from the same participants prior to G-CSF treatment, making it impossible to analyze pregnancy outcomes within this study design. Using a random-effects model, the meta-analysis revealed a significantly increased EMT in the G-CSF group compared to the control group (Mean differences [MD] = 1.79, 95% CI: 0.92–2.67). Additionally, the implantation rate (RR = 2.35, 95% CI: 1.20–4.60) and clinical pregnancy rate (RR = 2.52, 95% CI: 1.39–4.55) were significantly higher in the G-CSF group. These results support the efficacy of intrauterine G-CSF infusion in improving both EMT and pregnancy outcomes. However, the study acknowledged limitations, including a small sample size and the lack of robust randomization in the included trials (Xie et al., 2017).

More recently, in 2023, Fu et al. conducted a systematic review and meta-analysis exclusively analyzing RCTs to evaluate the efficacy of G-CSF in infertile women undergoing IVF treatment. A total of 20 RCTs were included, with the majority (14 studies) focusing on women with RIF, while only three studies specifically targeted patients with a history of thin endometrium. Subgroup analysis of the “thin endometrium” cohort revealed a significantly higher clinical pregnancy rate in the G-CSF group compared to the control group (RR = 1.85, 95% CI: 1.07–3.18). Additionally, EMT was significantly increased following G-CSF treatment (MD = 2.25, 95% CI: 1.58–2.92), although only two studies reported EMT data (Fu et al., 2023).

A review of the literature on granulocyte colony-stimulating factor (G-CSF) treatment for thin endometrium reveals key insights into its administration, effectiveness, and clinical outcomes.

One of the most notable findings is the absence of a standardized treatment protocol. Studies vary widely in dosage, timing of administration, reassessment intervals, and whether the treatment is applied in fresh or frozen embryo transfer (FET) cycles. However, intrauterine infusion remains the predominant route of administration across studies, suggesting a general consensus on its effectiveness in delivering G-CSF directly to the endometrium.

In cohort and self-controlled studies, G-CSF treatment has been associated with a significant increase in endometrial thickness. Despite this improvement, some patients fail to reach the commonly accepted threshold of 7 mm for optimal implantation, highlighting the variability in individual responses. Controlled studies have produced inconsistent results. Non-randomized trials report no significant differences in endometrial thickness or pregnancy outcomes, whereas randomized controlled trials (RCTs) demonstrate notable improvements in both parameters. This discrepancy underscores the need for further rigorous investigation to clarify G-CSF’s true efficacy. Interestingly, clinical pregnancy rates tend to be higher in FET cycles compared to fresh embryo transfers following G-CSF treatment. This aligns with the broader IVF consensus that FET cycles provide a more favorable uterine environment by avoiding the potential negative effects of controlled ovarian stimulation. These findings suggest that G-CSF may be particularly beneficial for specific patient populations, especially those undergoing FET cycles.

Overall, while G-CSF shows promise as a potential therapy for thin endometrium, significant heterogeneity exists in study protocols and outcomes. The inconsistent findings across different study designs underscore the need for well-designed, large-scale RCTs to establish optimal treatment protocols, determine patient selection criteria, and clarify the true clinical benefits of G-CSF in assisted reproductive technologies (Table 3).

## 4 Growth hormone (GH) therapy and its impact on endometrial function

GH is a peptide hormone secreted by the anterior pituitary gland, widely recognized for its role in growth and metabolism. While early research on GH in reproductive medicine primarily focused on its effects on oocyte quality and ovarian response, its potential influence on endometrial function has received relatively less attention. However, emerging evidence suggests that GH plays a significant role in endometrial proliferation, vascularization, and implantation (Chang et al., 2022). GH receptors have been identified in rat endometrium and human myometrium, indicating a potential role in uterine function (Altmäe and Aghajanova, 2019). Moreover, in human endometrial tissue, GH receptors are expressed in glandular epithelial cells, with peak expression observed during the mid-to-late luteal phase and in decidual tissue during pregnancy. This temporal pattern suggests that GH may be closely involved in the implantation process and endometrial receptivity (Sbracia et al., 2004). The biological effects of GH on the endometrium are primarily mediated through its ability to upregulate key growth factors and signaling molecules involved in endometrial remodeling. One of these factors is insulin-like growth factor-1 (IGF-1), which mediates GH to promote endometrial cell proliferation and differentiation. Additionally, GH enhances the expression of vascular endothelial growth factor (VEGF), a crucial regulator of angiogenesis that improves blood supply to the endometrium. Furthermore, GH has been shown to regulate the expression of integrin β3, a key marker of endometrial receptivity that plays an essential role in embryo implantation (Cui et al., 2019). These molecular mechanisms suggest that GH supplementation may facilitate endometrial development, particularly in women with thin endometrium or recurrent implantation failure.

Several clinical reports and trials suggest that GH supplementation may be beneficial for women experiencing RIF or persistently thin endometrium. A case report highlighted a patient with panhypopituitarism who had repeated implantation failures due to inadequate endometrial thickness. The addition of the GH to her treatment protocol successfully enhanced endometrial development, ultimately resulting in a successful pregnancy (Drakopoulos et al., 2016). Furthermore, multiple clinical trials have demonstrated that GH administration can improve endometrial thickness, upregulate endometrial receptivity markers, and enhance pregnancy outcomes (Table 4). These findings further support the potential therapeutic role of GH in ART.

**TABLE 4:** Growth hormone therapy for refractory thin endometrium.

Study	Objective	Samples	ET protocol	GH administration [Timing]	Results (GH vs control)
Original article not available (data from reviews and meta-analysis) (Altmäe and Aghajanova, 2019; Liu et al., 2020; Shang et al., 2022)					
Huang (2011) RCT	Thin EM	GH: 62 Control: 52	FET (HRT)	4 IU/d SC [D8 of HRT ∼ EMT >7 mm]	EMT: 7.9 ± 1.18 vs 6.6 ± 1.54 CPR: 45.2% vs 19.2%
Xiang (2011) RCT	Thin EM	GH: 32 Control: 35	Fresh ET (long protocol)	4.5 IU/d SC [D3 ∼ hCG day]	CPR: 37.5% vs 14.3%
Li (2015) RCT	Thin EM	GH: 42 Control: 38	FET (HRT)	5 IU/d SC [D3 of HRT ∼ P start]	EMT: 8.04 ± 2.27 vs 6.57 ± 2.01 CPR: 50% vs 39.5%
Wu (2013) Prospective cohort	Thin EM	GH: 32 Control: 30	FET (HRT)	4 IU/d SC [∼hCG day]	Significantly thicker EMT in the GH group EMT: 8.8 ± 1.3 vs 7.1 ± 1.9 (p < 0.05)
Ling (2016) Retrospective cohort	Thin EM (EMT<7 mm)	35	FET (HRT)	4 IU/d [D3-5 of HRT ∼ P start day]	Improved EMT and CPR
Mei (2018) RCT	Thin EM (EMT<8 mm)	63	NM	4 IU/d for 10 days	Improved EMT and CPR
Original article available					
Yu et al. (2016) Prospective cohort	Thin EM	5	FET (HRT)	6 IU/intrauterine infusion 4–5 times [D9∼D12 of HRT]	Increased EMT before and after treatment EMT: 5.78 ± 0.65 vs7.96 ± 0.71 (p < 0.05)
Cui et al. (2019) RCT	History of fresh ET cycle cancellation due to EMT ≤ 7 on hCG day	GH: 40 Control: 53	FET (HRT)	5 IU/d SC [D3∼D18 of HRT]	Significant higher IR and CPR in the GH group IR: 24.4% vs. 10.5% (p < 0.001) CPR: 43.6% vs. 19.2% (p = 0.012)
Yang et al. (2019) Retrospective cohort	EMT <8 mm on the P start day	225 (GH: 184 cycles) (Control: 61 cycles)	FET (HRT)	4.5 IU SC every alternate day [P start day ∼ ET day]	Significant higher IR and CPR in the GH group IR: 44.8% vs 32.8% (p = 0.019) CPR: 64.7% vs 49.2% (p = 0.032)
Hosseini et al. (2022) Prospective cohort	History of cycle cancellations due to EMT <7 mm; EMT < 7 mm on D13 of HRT	31	HRT	5 mg/intrauterine infusion max 5 times [D14 of HRT ∼ EMT ≥ 7 mm]	Significantly increased EMT before and after treatment EMT: 5.14 ± 1.11 vs 7.03 ± 1.23 (p < 0.001) 11/31 (35.5%) EMT did not reach 7 mm CPR: 7/20, 35%
Wu et al. (2023) RCT	History of fresh ET cycle cancellation due to thin EM	GH: 19 Control: 19	FET (HRT)	SC	Significantly thicker and higher growth rate of EMT in the GH group (p < 0.05) Slightly higher CPR in GH group: 36.8% vs 31.6%

Abbreviations: GH, growth hormone; RCT, randomized controlled trial; EM, endometrium; FET, frozen embryo transfer; HRT, hormone replacement therapy; IU, international unit; SC, subcutaneous injection; EMT, endometrial thickness; CPR, clinical pregnancy rate; ET, embryo transfer; NM, not mentioned; IR, implantation rate; P, progesterone.

### 4.1 Randomized controlled trials

Several RCTs have been conducted, demonstrating the potential effects of GH on endometrial expansion and improving pregnancy outcomes (Table 4).

One notable RCT, published by Cui et al., in 2019, investigated GH supplementation in patients with a history of fresh embryo transfer (ET) cycle cancellation due to thin endometrium. The study included 93 patients, who were randomly assigned to either the GH group (n = 40) or the control group (n = 53). In addition to the hormone replacement therapy (HRT), patients in the GH group received subcutaneous GH injections (5 IU per day) from day 3 to day 18, whereas the control group received the HRT alone. At the time of reassessment, 10 patients in the GH group and 25 in the control group still had an endometrial thickness (EMT) below 7 mm. The results indicated that GH treatment significantly improved baseline EMT (7.87 ± 0.72 vs 6.34 ± 0.86), implantation rates (24.4% vs 10.5%, p < 0.001), and clinical pregnancy rates (43.6% vs 19.2%, p = 0.012) compared to the control group. Additionally, *in vitro* experiment revealed that GH significantly upregulated the expression of VEGF, integrin β-3, and IGF-1 at both mRNA and protein levels in endometrial cells, suggesting that GH enhances endometrial thickness and receptivity through angiogenesis, cell proliferation, and estrogen-mediated effects (Cui et al., 2019).

A more recent RCT by Wu et al., published in 2023, examined a smaller cohort of 38 participants with a history of fresh ET cycle cancellation due to thin endometrium. Participants were randomly assigned to either the GH group (n = 19) or the control group (n = 19), with GH administered *via* subcutaneous injection. The study reported significantly increased EMT and endometrial growth rates in the GH group compared to the control group. However, while a trend toward improved clinical pregnancy rates was observed, the difference was not statistically significant (Wu et al., 2023).

These RCTs suggest that GH supplementation may be a promising strategy to enhance endometrial development and improve pregnancy outcomes, particularly in women with persistently thin endometrium. However, further large-scale trials are needed to establish standardized treatment protocols and confirm its clinical efficacy.

### 4.2 Luteal phase GH injection

While most clinical trials have administered GH during the early or mid-follicular phase, Yang et al. explored the effects of GH supplementation during the early luteal phase. In this retrospective study, GH injection (4.5 IU subcutaneously) was administered every other day, starting alongside progesterone during the early luteal phase and continuing until the embryo transfer. A total of 225 women were included in the study, with GH treatment applied in 184 cycles and standard HRT used in the remaining 61 cycles. The results demonstrated a significantly higher clinical pregnancy rate in the GH group compared to the control group (64.7% vs 49.2%, p = 0.032), suggesting that luteal-phase GH administration may enhance endometrial receptivity and improve implantation outcomes. The underlying mechanism may be linked to GH receptor expression in the glandular cells of the endometrium during the luteal phase, where GH could facilitate implantation through its regulatory effects on endometrial remodeling and receptivity (Yang et al., 2019).

This study provides a novel perspective on GH administration timing, indicating that luteal-phase GH supplementation may be a valuable approach for improving pregnancy outcomes in ART. However, further randomized controlled trials are needed to validate these findings and optimize treatment protocols.

### 4.3 Intrauterine infusion of GH

Beyond subcutaneous GH administration, some studies have explored the intrauterine infusion of GH as an alternative approach. This localized delivery method is thought to offer advantages over systemic administration by directly targeting endometrial cells, potentially enhancing endometrial response while minimizing systemic side effects.

The first report on intrauterine GH infusion was a small single-arm study involving five patients. These participants received intrauterine GH infusions (6 IU per dose) from days 9–12 of the hormone replacement therapy (HRT) cycle, with a total of four to five infusions. By the day of progesterone administration, the mean endometrial thickness (EMT) had significantly increased to 7.96 ± 0.71 mm, compared to 5.78 ± 0.65 mm on the first day of infusion (Yu et al., 2016).

A subsequent prospective study evaluated the effects of intrauterine GH infusion (5 mg per dose) administered from day 14 of the HRT cycle until the EMT reached 7 mm, with a maximum of five infusions. The study included 31 participants with a history of multiple cycle cancellations due to persistently thin endometrium (<7 mm) despite standard HRT. The mean EMT increased significantly from 5.14 ± 1.1 mm to 7.03 ± 1.23 mm after GH treatment. However, in 11 participants (35.5%), the EMT remained below 7 mm, leading to embryo transfer (ET) cancellation. Among the 20 participants who proceeded with ET, the clinical pregnancy rate was 35% (7/20). Notably, the study found a significant correlation between EMT on ET day and pregnancy outcomes. Women who achieved clinical pregnancy had significantly thicker endometrial linings on the ET day compared to those who did not conceive (7.18 ± 0.56 mm vs 6.21 ± 0.72 mm, p = 0.007) (Hosseini et al., 2022).

These findings suggest that intrauterine GH infusion may be a promising strategy for women with refractory thin endometrium, potentially improving endometrial development and pregnancy rates. However, larger randomized controlled trials are needed to further establish their efficacy and refine treatment protocols.

### 4.4 Meta-analysis and reviews

Several reviews and meta-analyses have evaluated the effects of GH on endometrial thickness and pregnancy outcomes in infertile women undergoing IVF. Altmäe et al. and Liu et al. reviewed 4 and 6 comparative studies, respectively, conducted between 2013 and 2019, with 4 overlapping studies included in both reviews (Table 4). These studies evaluated the use of GH administration in HRT cycles for FET. Despite variations in treatment protocols, the overall findings supported the beneficial effects of GH on endometrial thickness, receptivity, and pregnancy outcomes (Altmäe and Aghajanova, 2019; Liu et al., 2020).

A 2022 meta-analysis by Shang et al. included 4 RCTs with a total of 354 patients diagnosed with thin endometrium (Table 4). The GH dosage in these studies ranged from 4 to 5 IU per day, administered subcutaneously from day 3 to day 8 of the ovarian stimulation or HRT cycle. The pooled data indicated a significant benefit of GH supplementation, with improvements in clinical pregnancy rate (Odds Ratio, OR = 2.71, 95% CI: 1.69–4.34; P < 0.0001), endometrial thickness (MD = 1.48, 95% CI: 1.21–1.75; P < 0.00001), and endometrial receptivity (Shang et al., 2022). In 2021, Jiang et al. conducted a comparative study to evaluate FET outcomes in patients with thin endometrium following treatment with either G-CSF or GH. The study included 66 patients who had failed their first fresh embryo transfer cycle, with 25 assigned to the G-CSF group and 41 to the GH group. The G-CSF group received intrauterine infusions of 1 mL G-CSF on the third, fifth and seventh days of the HRT cycle, while the GH group received daily subcutaneous GH (5 IU/day) from the start of HRT until progesterone administration. Endometrial monitoring showed that the GH group had significantly greater endometrial thickness on the fifth day of HRT (5.75 ± 1.27 vs 4.83 ± 0.85 mm, p = 0.001); however, no significant differences were observed on later monitoring days or at the time of progesterone administration. Additionally, the implantation rates (G-CSF, 34.8%; GH, 27.5%), clinical pregnancy rates (G-CSF, 52%; GH, 46.3%), and live birth rates (G-CSF, 40%; GH, 31.7%) were comparable between the two groups. The study underscored the clinical value of both treatments, as pregnancy rates increased by 10%–20% in treated patients compared to those who had not received any endometrial intervention at the same center (Jiang et al., 2021).

Although no standardized GH treatment protocol for thin endometrium exists, most studies consistently report its beneficial effects on endometrial thickening and pregnancy outcomes (Table 5). Early trials primarily administered GH *via* daily subcutaneous injections during the early to mid-follicular phase. More recent studies have explored alternative methods, such as intrauterine infusion and luteal-phase administration, with promising results. However, no conclusive evidence has yet determined the most effective route or timing for GH administration, highlighting the need for further well-designed trials.

**TABLE 5:** Stem cell therapy for Asherman syndrome and/or thin endometrium.

Study	Objective	Samples	Autologous stem cell origin	Administration route	EMT change (mm)	Outcomes
Nagori et al. (2011)	AS with thin EM	1	Endometrial angiogenic stem cells (BM aspiration)	EM scratching + Intrauterine infusion	From 3.6 to 7.1	• Clinical pregnancy via IVF, followed until 8 weeks
Singh et al. (2014)	AS	6	Mononuclear stem cells CD34^+^ (BM aspiration)	Sub-endometrial injection (trans- myometrium)	Significant increase Pre-treatment: 1.38 ± 0.39 3 months: 4.05 ± 1.40 (p < 0.05) 6 months: 5.46 ± 1.36 (p = 0.001) 9 months: 5.48 ± 1.14 (p = 0.002)	• 5/6 resume of menstrual cycle
Santamaria et al. (2016)	AS and EA (EMT <5 mm)	16 (AS 11; EA 5)	Mononuclear stem cells CD133+ (Peripheral blood)	Spiral arterioles through intra-arterial catheterization	EA group: significant increase Pre-treatment (max): 4.2 Post-treatment (max): 5.7 (p = 0.03)	• 15/16 resume of menstrual cycle • 3 spontaneous conceptions: 1 live birth • 14 ET: 7 pregnancies; 1 live birth
Tan et al. (2016)	AS	7	Menstrual blood-derived stromal cells	EM scratching + Intrauterine infusion	5/7 EMT >7 mm	• 1 spontaneous conception • 5 ET: 2 pregnancies
Sudoma et al. (2019)	Thin EM (<5 mm) with ≥ 3 IVF failures	25	Adipose-derived stem cells	Sub-endometrial injection (trans-myometrium); 3 times	20/25 (80%) increase EMT at 6 months	• 2 spontaneous conceptions • 19 ET: 11 pregnancies • Total 9 live births
Sapozhak et al. (2020)	Thin EM	1	Endometrial mesenchymal stem cells	Hysteroscopic-guide submucosal injection	From 2 to 4–6.3 mm	• ET (2 embryos): a twin pregnancy, 1 take-home baby
Singh et al. (2020)	AS and EA (EMT <5 mm)	25 (AS 12; EA 13)	Mononuclear stem cells (BM aspiration)	Sub-endometrial injection (trans-myometrium)	Significant increase in 3 months Pre-treatment: 3.3 ± 1.0 3 months: 5.1 ± 1.9 (p = 0.001) 6 months: 5.6 ± 1.5 (p = 0.164) 9 months: 6.1 ± 1.7 (p = 0.135)	• 6/7 resume of menstrual cycle • 2 spontaneous conceptions: 2 live births • 11 ETs: 1 live birth
Tersoglio et al. (2020)	Thin EM (<7 mm) with RIF	29	Endometrial mesenchymal stem cells, diluted with autologous PRP	Sub-endometrial injection (trans-myometrium)	Significant increase Pre-treatment: 5.24 ± 1.24 Post-treatment: 9.93 ± 0.77	• 29 ET • CPR: 23/29 (79.31%) • LBR: 10/22 (45.45%)
Zhang et al. (2021)	Thin EM (<5.5 mm) caused by AS	17	Umbilical cord mesenchymal stem cells coated on collagen scaffolds	Scaffolds spread onto the Foley catheter, placed in the uterine cavity with a filled balloon; 2 times	Significant increase Pre-treatment: 4.08 ± 0.26 3 months: 5.87 ± 0.77 (p < 0.001)	• 1 spontaneous conception: 1 live birth • 22 ETs: 3 pregnancies, 2 live births

Abbreviations: EMT, endometrial thickness; AS, Asherman syndrome; EM, endometrium; BM, bone marrow; IVF, *in vitro* fertilization; EA, endometrial atrophy; ET, embryo transfer; RIF, recurrent implantation failure; PRP, platelet rich plasma; CPR, clinical pregnancy rate; LBR, live birth rate.

## 5 Stem cell therapy

Stem cell-based therapies have emerged as a promising approach for endometrial regeneration, particularly in cases of thin endometrium and Asherman’s syndrome. Stem cells derived from various tissues—including bone marrow, endometrium, adipose tissue, and umbilical cord—have demonstrated regenerative potential and the ability to differentiate into multiple cell types (Nagori et al., 2011). Extensive research in basic science and animal models has provided insights into the underlying mechanisms of stem cell-mediated endometrial repair, including the identification of key markers and differentiation pathways (Gharibeh et al., 2022). Among the different stem cell sources, bone marrow-derived stem cells (BMSCs) and endometrial-derived stem cells (EnSCs) are the most commonly studied in clinical applications for endometrial regeneration. These cells are believed to contribute to endometrial repair through multiple mechanisms: direct differentiation into endometrial epithelial or stromal cells, activation of resident endometrial stem cells, and recruitment of circulating stem cells from other tissues (Singh et al., 2014; Abuwala and Tal, 2021; Sapozhak et al., 2020).

Clinical studies have explored various stem cell delivery methods for endometrial regeneration, including sub-endometrial injection for direct integration, intrauterine infusion to facilitate migration into the tissue, and spiral arteriole injection to enhance blood supply (Table 5). Each approach aims to improve endometrial repair and function, potentially benefiting patients with thin endometrium or endometrial damage. Several review articles have explored the potential of stem cell therapy in endometrial repair, highlighting the promising outcomes observed in both preclinical and clinical studies (Table 5). However, challenges remain, including the need for standardized protocols, optimization of cell sources and dosages, and long-term safety assessments.

### 5.1 Bone marrow-derived mesenchymal stem cells

Nagori et al. reported a case of successful stem cell therapy for severe Asherman syndrome with refractory thin endometrium. The patient, a 33-year-old woman, had repeatedly failed FET cycles due to persistently thin endometrium (<3.6 mm) despite undergoing endometrial adhesiolysis and multiple hormone therapy cycles. Endometrial angiogenic stem cells were isolated from the patient’s bone marrow *via* aspiration and infused into the uterine cavity following endometrial curettage. Subsequent hormone therapy facilitated endometrial expansion to 7.1 mm within 4 months, allowing for successful donor oocyte transfer. The patient conceived, with pregnancy confirmed up to 8 weeks of follow-up (Nagori et al., 2011).

Singh et al. and Santamaria et al. explored the use of mononuclear stem cells, derived from bone marrow and peripheral blood, respectively, for endometrial regeneration (Singh et al., 2014). Singh et al. recruited six women with Asherman syndrome and secondary amenorrhea, primarily caused by genital tuberculosis. CD34-positive endothelial progenitor cells—known for their role in angiogenesis and tissue repair—were transplanted into sub-endometrial zone *via* transvaginal ultrasound-guided injection. Three months post-treatment, the mean EMT significantly increased from 1.38 to 4.05 mm (p = 0.008), though further improvements plateaued at six and 9 months. Notably, 5 out of six women resumed their menstrual cycles (Singh et al., 2014).

Santamaria et al. investigated a cohort of 16 women (11 with Asherman syndrome and 5 with atrophic endometrium, EMT <5 mm). CD133-positive stem cells were administered *via* intra-arterial catheterization into the spiral arterioles. In patients with atrophic endometrium, EMT significantly increased from 4.2 to 5.7 mm (p = 0.03). Menstrual cycles resumed in 15 of 16 women, and 3 conceived spontaneously, leading to one live birth. A total of 14 embryo transfers were performed, resulting in seven pregnancies and one additional live birth (Santamaria et al., 2016). Years later, Singh et al. conducted a larger prospective, single-arm study using the same stem cell transplantation method. The study enrolled 25 patients (12 with Asherman syndrome and 13 with atrophic endometrium, defined as an EMT of less than 5 mm). The findings, which align with prior results, show significant EMT improvement over the 3 months (from 3.3 mm to 5.1 mm, p = 0.001), with only marginal increases at six and 9 months. Eleven women achieved an EMT of ≥7 mm and proceeded with IVF treatment. During a 5-year follow-up, two women conceived spontaneously at 3 years post-treatment, while another conceived *via* IVF at 4 years post-treatment. All three pregnancies resulted in live birth (Singh et al., 2020).

### 5.2 Stem cells from alternative sources

Beyond bone marrow-derived stem cells, stem cells harvested from various tissues, including menstrual blood, endometrial tissue, adipose tissue, and umbilical cord, have been explored as potential therapies for infertile women with thin endometrium (Table 5).

Sapozhak et al. and Tersoglio et al. investigated the use of endometrial mesenchymal stem cells obtained *via* endometrial biopsy. These were delivered to the sub-endometrial zone through hysteroscopic-guided and trans-myometrium injection, respectively. Sapozhak et al. reported the case of a 38-year-old woman with persistently thin endometrium (<4 mm) despite multiple hormone therapy cycles. After endometrial mesenchymal stem cells therapy, her EMT increased to 6.3 mm, enabling the transfer of two embryos. She ultimately achieved a twin pregnancy, resulting in one premature birth and one healthy take-home baby (Sapozhak et al., 2020). Tersoglio et al. conducted a study on 29 women with a thin endometrium (EMT <7 mm) and RIF. In addition to the stem cells, autologous PRP was used as a diluent to enhance stem cell function. The therapy led to a significant increase in mean EMT (from 5.24 to 9.93 mm), with all participants proceeding to embryo transfer. The clinical pregnancy rate was 79.31% (23/29), and the live birth rate reached 45.45% (10/22) (Tersoglio et al., 2020).

Tan et al. explored the use of menstrual blood-derived stromal cells in treating infertile women with severe Asherman syndrome. Menstrual blood was collected *via* catheter on day 2 of the cycle, rinsed with penicillin/streptomycin, and processed for stem cell isolation. The stem cell suspension was then infused into the uterine cavity after endometrial scratching on day 16 of the same menstrual cycle. Five out of seven women achieved an EMT of at least 7 mm, suitable for embryo transfer. One woman conceived spontaneously, while two others achieved pregnancy *via* IVF (Tan et al., 2016).

Sudoma et al. investigated the use of autologous adipose-derived stem cells (ADSCs) for women with thin endometrium (EMT <5 mm) and at least three prior IVF failure. Adipose tissue was aspirated from the abdominal wall, and stem cells were isolated based on CD34 positivity (vascular endothelial marker) and CD45 negativity (lymphohematopoietic marker). The ADSCs were injected into the sub-endometrial zone through the myometrium in three sessions, spaced 5–7 days apart, under an artificial cycle. Within 6 months, 80% (20/25) of participants experienced an increase in EMT. Two conceived spontaneously, and among 19 embryo transfers, 11 resulted in pregnancy (57.7%), leading to nine live births (Sudoma et al., 2019).

Zhang et al. conducted a pilot study using collagen scaffolds loaded with umbilical cord mesenchymal stem cells to treat patients with thin endometrium (EMT <5.5 mm) caused by Asherman syndrome. The collagen scaffold, designed to promote cell adhesion and growth, was coated with UC-MSCs and placed onto a Foley catheter. The catheter was then inserted into the uterine cavity, with its balloon filled to facilitate scaffold attachment to the endometrial lining. The Foley balloon remained in place for 3 days before the removal. Seventeen patients underwent this procedure across two consecutive menstrual cycles. Three months post-treatment, EMT significantly increased (from 4.08 mm to 5.87 mm), although endometrial volume, vascularization, and uterine artery blood flow showed no significant improvement. One patient conceived spontaneously, leading to one live birth. Among 15 patients who underwent 22 embryo transfers, three pregnancies and two live births were achieved (Zhang et al., 2021).

Currently, several case reports, case series, and cohort studies have demonstrated the potential of stem cell therapy for Asherman syndrome and thin endometrium in infertile women (Table 5). However, no randomized controlled trials or large-scale comparative studies have been published, making it difficult to establish standardized treatment protocols or determine the long-term efficacy and safety of stem cell-based interventions. Notably, patients undergoing stem cell therapy often present with more severe endometrial conditions, such as persistent refractory thin endometrium or extensive intrauterine adhesions, compared to those treated with PRP, G-CSF, or GH. These alternative therapies are often used as first-line regenerative treatments, while stem cell therapy is considered for patients with more advanced pathology who have failed standard approaches. Despite the severity of cases treated with stem cell therapy, most studies report consistent and promising outcomes, including significant endometrial expansion, restoration of menstrual cycles, and improved pregnancy success rates (Table 5). However, further research is needed to compare the effectiveness of different stem cell sources, optimize delivery methods, and assess long-term reproductive outcomes and potential risks associated with stem cell transplantation.

## 6 Exosomes derived from stem cells

Exosomes are nanoscale extracellular vesicles secreted by various cell types, including stem cells. These vesicles contain a diverse cargo of bioactive molecules such as proteins, messenger ribonucleic acids (mRNAs), microRNAs (miRNAs), and lipids, facilitating intercellular communication and molecular transport. Compared to stem cells, exosomes are considered to have superior biological safety, a lower risk of immune rejection, and reduced potential for tumorigenicity, making them an attractive candidate for therapeutic applications (Liu et al., 2024; Tabeeva et al., 2023).

Recent studies have increasingly focused on the role of stem cell-derived exosomes in endometrial repair and regeneration. Currently, most research has been conducted in animal models, utilizing both *in vitro* and *in vivo* approaches. However, the majority of these studies have primarily examined exosomes in the context of endometrial injury rather than explicitly addressing thin endometrium. To bridge this gap, our research expands the focus to include both injured and thin endometrial conditions.

Emerging evidence suggests that stem cell-derived exosomes promote endometrial regeneration by enhancing tissue repair, stimulating neo-vascularization, exerting anti-fibrotic effects, and improving endometrial receptivity and fertility (Liu et al., 2024; Tabeeva et al., 2023). Although the precise mechanisms are still being elucidated, several key pathways have been identified. These include the suppression of fibrotic signaling, downregulation of inflammatory responses, immunomodulation, and collagen remodeling. Future studies should aim to further clarify the molecular mechanisms underlying these effects and explore the potential for clinical translation in treating endometrial dysfunction.

### 6.1 Mechanism of exosome-mediated endometrial repair and regeneration

In animal models, Yao et al. discovered that exosomes derived from bone marrow stem cells (BMSCs) play a significant role in repairing injured endometrium by downregulating TGF-β/Smad signaling pathway. This pathway is closely associated with epithelial-mesenchymal transition (EMT) and endometrial fibrosis. After treatment with BMSCs and BMSCs-derived exosomes, significant improvements were observed, including an increased number of endometrial glands and a reversal of endometrial fibrosis (Y et al., 2019). *In vitro* studies have also demonstrated the potential of exosomes in mitigating endometrial injury. For example, an injury model was created by exposing mouse endometrial epithelial cells to conditions of oxygen-glucose deprivation followed by reoxygenation. After treatment with human umbilical cord mesenchymal stem cell (UCMSC)-derived exosomes, cell viability, which had significantly decreased under injury conditions, was notably restored. The mechanism behind this was linked to the exosome-mediated reduction of pro-inflammatory cytokines, such as IL-6, IL-1β, and TNF-α, through the Toll-like receptor 4 (TLR4) signaling pathway (Liang et al., 2020).

Additionally, Xin et al. investigated the immunomodulatory effects of UCMSC-derived exosomes *in vivo*. Exosomes were loaded onto the collagen scaffold and transplanted onto the injured endometrial surface of rats. The results showed that the regenerated endometrium was significantly thicker, exhibited enhanced neovascularization, and showed increased expression of estrogen and progesterone receptors. The study also demonstrated that UCMSC-derived exosomes could influence macrophage polarization toward an anti-inflammatory phenotype (M2 macrophages), thereby increasing the levels of anti-inflammatory cytokines, such as IL-10, TGF-β1, and VEGFB (Xin et al., 2020). Wang et al. identified that one of the most enriched microRNAs in exosomes derived from human UCMSCs, miRNA-202-3p, plays a critical role in the early stages of endometrial repair. This microRNA targets the matrix metallopeptidase 11 (MMP11) gene, thereby regulating the remodeling of the extracellular matrix (ECM) and facilitating tissue regeneration (Wang et al., 2023).

### 6.2 Exosomes combined with biomaterial scaffolds for enhanced endometrial regeneration

In recent years, several studies have focused on combining exosomes derived from various stem cells with biomaterial scaffolds, including hyaluronic acid, collagen, and hydrogels. These scaffolds mimic the structure of the extracellular matrix, promoting tissue proliferation, migration, and regeneration (Lin et al., 2021). Below are examples of studies demonstrating the potential of exosome-biopolymer combinations for endometrial regeneration, improved endometrial receptivity, and fertility restoration.

### 6.3 Exosome-hydrogel scaffolds for endometrial regeneration

Lin et al. loaded the adipose stem cell (ADSC)-derived exosomes onto a hydrogel scaffold to treat rats with ethanol-induced endometrial damage. Following the intrauterine injection of the ADSC-derived exosome-hydrogel combination, significant endometrial regeneration was observed. This was evidenced by the presence of well-distributed cells, increased neovascularization, and elevated expression of markers associated with endometrial receptivity, including VEGF, LIF, avβ3, and IGF-1 (Lin et al., 2021).

### 6.4 Hyaluronic acid-based gel with UCMSC-derived exosomes

In another study, UCMSC-derived exosomes were mixed with a gel composed of hyaluronic acid. After intrauterine injection, the regenerative endometrium was significantly thicker, with increased gland number and micro-vessel density. Notably, markers associated with endometrial receptivity and implantation rates were significantly higher, indicating enhanced fertility potential (Zhang et al., 2022).

### 6.5 Alginate-based hydrogel for endometrial repair

Liang et al. encapsulated exosomes derived from endometrial decidual stromal cells in an alginate-based hydrogel. Post-treatment results showed positive effects on endometrial regeneration, including an increase in endometrial thickness and the number of glands. Moreover, angiogenesis, endometrial receptivity, and fertility recovery were significantly enhanced, suggesting the potential for this approach in treating thin endometrium (Liang et al., 2024).

### 6.6 Artificial mucus and urine-derived exosomes for endometrial repair

Li et al. derived exosomes from rat urine and combined them with an artificial mucus composed of hyaluronic acid and synthesized estrogenic substance. Treatment in a thin endometrium model led to a significant increase in endometrial thickness, receptivity, and fertility. Moreover, similar therapeutic effects were observed with exosomes derived from human urine, human ADSCs, and rat ADSCs, demonstrating the versatility of the artificial mucus system for endometrial regeneration (Li et al., 2024). These studies highlight the promising therapeutic potential of exosomes, particularly when combined with biomaterial scaffolds, for the regeneration of endometrial tissue, enhancement of endometrial receptivity, and restoration of fertility. The continued investigation of exosome-loaded scaffolds holds great promise for clinical applications in the treatment of endometrial dysfunctions, including thin endometrium and infertility.

## 7 Expanded context on therapeutic effects beyond thin endometrium

Beyond their role in promoting endometrial regeneration, regenerative therapies have also demonstrated beneficial effects in other infertility-related conditions, highlighting their broader potential in reproductive medicine. Integrating these findings provides a more comprehensive view of how such interventions may enhance IVF outcomes through multiple biological pathways. For example, PRP has been shown to enhance ovarian function and improve oocyte maturation and fertilization capacity, with clinical evidence demonstrating improved reproductive outcomes in women with diminished ovarian reserve (Elias et al., 2024; Sills et al., 2018) and experimental data confirming beneficial effects on immature oocyte maturation in mice (Ullo et al., 2015). G-CSF has been reported to improve implantation and pregnancy rates in recurrent implantation failure and unexplained infertility (Scarpellini and Sbracia, 2009; Aleyasin et al., 2016). GH has been associated with improved ovarian response, enhanced embryo quality, and increased live birth rates in poor ovarian responders (Chang et al., 2022; Yang et al., 2020). Stem cell therapy has demonstrated regenerative effects not only in endometrial repair but also in restoring ovarian function in premature ovarian insufficiency models (Liu et al., 2014; Shareghi-Oskoue et al., 2021). Finally, exosomes derived from stem cells have been shown to promote follicular development, granulosa cell proliferation, and oocyte competence in experimental systems (Zhou et al., 2024). These expanded discussions provide readers with a broader translational perspective on how regenerative therapies may benefit multiple infertility-related conditions and complement conventional IVF approaches.

## 8 Limitations of this narrative review

As a narrative review, this article does not follow a systematic methodology with predefined search strategies, inclusion/exclusion criteria, or formal quality assessment, which may limit reproducibility and transparency. The literature discussed encompasses case reports, case series, cohort studies, randomized trials, and meta-analyses, but heterogeneity in study design, sample size, and outcome measures make direct comparison challenging. Additionally, many studies are limited by small cohorts and variable protocols, highlighting the need for larger, well-designed randomized controlled trials to validate the clinical efficacy and safety of these regenerative therapies.

## 9 Conclusion

In recent years, regenerative therapies for thin endometrium have been extensively explored through various clinical studies, including case reports, case series, cohort studies, and controlled trials. Several meta-analyses have also been conducted to synthesize findings; however, high-quality RCTs focusing specifically on populations with refractory thin endometrium remain scarce. Moreover, inconsistencies in findings across studies may be attributed to variations in study design, sample characteristics, and outcome measures. A major challenge in the field is the lack of standardized treatment protocols, including optimal dosages, administration timing, and delivery routes. Without uniform guidelines, translating promising experimental findings into clinical practice remains difficult. Nevertheless, regenerative approaches such as PRP, GH, G-CSF, and stem cell-based therapies have demonstrated potential benefits for infertile women with thin endometrium who do not respond to conventional hormonal or vasoactive treatments (Figure 1). Among these emerging therapies, exosome-based approaches derived from stem cells represent a particularly promising avenue. Exosomes offer a cell-free alternative with lower immunogenicity and tumorigenic risk compared to direct stem cell transplantation. Preclinical studies have demonstrated their ability to enhance endometrial regeneration through mechanisms such as modulating inflammatory responses, promoting angiogenesis, reducing fibrosis, and improving endometrial receptivity. Furthermore, the integration of exosomes with biomaterial scaffolds, such as hydrogels and collagen matrices, has shown potential to optimize their therapeutic efficacy by mimicking the extracellular matrix and enhancing local retention. Despite the promising preclinical data, the clinical translation of exosome-based therapies requires further investigation. Large-scale, well-designed RCTs are needed to establish their safety, efficacy, and long-term effects in human populations. Additionally, regulatory considerations, including standardization of exosome isolation, characterization, and quality control, must be addressed before these therapies can be widely implemented in clinical practice.

In conclusion, while regenerative therapies continue to evolve, exosome-based strategies hold great potential as novel and effective treatments for thin endometrium. Future research should focus on optimizing treatment protocols, elucidating precise mechanisms of action, and conducting rigorous clinical trials to validate their therapeutic benefits. If successfully translated to clinical applications, regeneration-based therapies could significantly improve reproductive outcomes for women with endometrial insufficiency, offering new hope for those struggling with infertility.
